# Experiences of people diagnosed with malaria regarding signs and symptoms at a selected village in the Vhembe District, Limpopo province, South Africa

**DOI:** 10.4102/curationis.v49i1.2757

**Published:** 2026-02-03

**Authors:** Wavhudi Kwinda, Aluwani D. Mudzweda, Takalani Luhalima

**Affiliations:** 1 Department of Advance Nursing Sciences, Faculty of Health Sciences, University of Venda, Thohoyandou, South Africa

**Keywords:** diagnosed, experiences, malaria, people, village

## Abstract

**Background:**

In 2021, there were about 263 million cases of malaria reported around the world and 579 reported deaths globally. There were extreme cases of malaria worldwide, regionally, provincially, and in the Vhembe District. Despite current efforts and several advancements to control vectors and prevent bites to minimise its burden, malaria remains a serious health issue.

**Objectives:**

The objective of the study was to explore the experiences of people diagnosed with malaria regarding signs and symptoms at Mhinga Village in the Vhembe District, Limpopo province, South Africa.

**Method:**

A qualitative method, with an exploratory, descriptive and contextual design was used to get an in-depth understanding of the phenomenon. Data were collected using semi-structured interviews with an interview guide focused on the experiences of people who were diagnosed with malaria regarding signs and symptoms at Mhinga Village in the Vhembe District, Limpopo. Participants were purposively selected. Qualitative thematic analysis was conducted using codes.

**Results:**

Two subthemes emerged: experiences with signs and symptoms and circumstances prompting immediate consultation. Different signs and symptoms were experienced based on the systems.

**Conclusion:**

The study concluded that the participants had experienced symptoms of malaria according to systems and certain circumstances were present that drove participants to consult immediately.

**Contribution:**

This study increased awareness of malaria prevention strategies, which may lower malaria transmission. Unique contributions include protecting Limpopo province’s Department of Health from admittance cuts. Malaria morbidity, death and treatment costs may decrease. Policymakers may encourage the community to continue malaria prevention methods, including mosquito avoidance and antimalarial use, which have been shown to work.

## Introduction

Malaria is an illness caused by the bite of the female Anopheles mosquito, and it was indicated that it can cause mortality in humans, especially children below the age of 5 years (Varo, Chaccour & Bassat 2020). Globally, Guo et al. (2021) found that some less malaria-prone countries, such as Germany, have been affected by malaria; people were diagnosed with falciparum malaria. In Tanzania, Lindgren and Persson (2020) documented cases where patients went through unnecessary delays in receiving the appropriate medical care because of misdiagnosis of malaria and therapy that varied with each stage of the disease. Lindgren and Persson (2020) also found that patients were administered intravenous fluids and antipyretics after experiencing fainting, unconsciousness, confusion, and fever. In 2021, there were about 247 million cases of malaria reported around the world (World Health Organization [WHO] 2022).

In Africa, malaria cases increased from approximately 218 million to 232 million, and deaths from 544 000 to 599 000 (WHO 2022). In Zimbabwe, those who presented with malaria signs and symptoms, such as vomiting, headache, and general body weakness, were tested on rapid diagnostic tests, and the results were positive for malaria (Takarinda et al. 2022). Varo et al. (2020) reported that in sub-Saharan Africa, children were predominantly diagnosed with malaria and died despite receiving appropriate treatment. Similarly, Adeyemo et al. (2022) indicated that low-income countries in sub-Saharan Africa have suffered significant economic losses and are unable to fund malaria intervention initiatives. According to Savi (2022), diseases and fatalities have occurred in sub-Saharan Africa, with an estimated 2000 children dying daily from the diagnoses. The malaria burden has been high in South Africa and has caused infections and deaths, particularly in areas where temperatures are high during the day and night (Sehlabana, Maposa & Boateng 2020). Moreover, malaria was diagnosed in people travelling to and from South Africa. In the Limpopo province of South Africa, malaria has remained endemic despite the annual implementation of a comprehensive and effective indoor residual spraying (IRS) programme targeting malaria vectors (Munzhedzi et al. 2021). Bornman et al. (2022) claimed that the Vhembe District has been suffering from malaria even following the yearly IRS performance. Gwarinda et al. (2021) further stated that Vhembe District is classified as a malaria transmission zone, contributing about 60% of the nation’s disease load. Tshiovhe et al. (2022) found that most families in the Vhembe District lived near streams, lakes, and dumping sites, which has increased malaria risks for them to be infected and diagnosed. Furthermore, it was stated that malaria infections have negatively affected the South African Department of Health financially because treatment has been expensive, and children who were diagnosed with malaria often died. The proposed study focused on the experiences of people diagnosed with malaria at Mhinga Village because there has been limited investigation about the experiences of people diagnosed with malaria in the Vhembe District of the Limpopo province.

## Rationale of the study

Gwarinda et al. (2021) focused on parasite genetic diversity, which reflected continued residual malaria transmission in the Vhembe District, a hotspot in the Limpopo province of South Africa. They found that Vhembe District remained a parasite transmission hotspot. Munzhedzi et al. (2021) studied community knowledge, attitudes and practices towards malaria in Ha-Lambani, Limpopo province, South Africa. They found that the knowledge of malaria prevention has been limited and there had been inadequate distribution of bed nets. There has been a knowledge gap in the experiences of people diagnosed with malaria in the villages of Vhembe District, and malaria has been causing death. If people become knowledgeable about malaria signs and symptoms, they will be able to take preventative measures and seek medical help early, which could reduce mortality and morbidity rates. The proposed study’s objective was to explore the experiences of people diagnosed with malaria regarding signs and symptoms at Mhinga Village in the Vhembe District.

### Problem statement

When one is diagnosed with malaria, treatment is provided in a hospital with prescribed drugs to kill the parasite, depending on the type of parasite, severity of symptoms and age. However, in the Mhinga community, people have been diagnosed with malaria infections, and deaths occurred with 105 cases from January to June 2023, according to the Vhembe District epidemiology office. This number was high compared to the neighbouring villages, suggesting that malaria has been an issue in Mhinga, as many people were still contracting the disease, and many of them lost their lives. Vhembe District hospital statistics have indicated that most people still experience and are being diagnosed with malaria even after several preventative measures, such as IRS, medical advancements, and awareness programmes, have been implemented. Mhinga Village is a flat area, and when it rains, water becomes stagnant for a long time, and female mosquitoes lay eggs in it and multiply. This has been problematic, as people were still being diagnosed and experiencing malaria at Mhinga Village even after prevention measures had been implemented. This has put a financial burden on the villagers and hospital systems. As a result, some community members have lost their loved ones and experienced psychological problems. The sustainable development goals (SDGs) are actions to transform the world so that people can prosper and enjoy peace. This study focuses on SDG goal number 3 and the agenda 2063, which aims for good health and well-nourished citizens, as well as promoting health, which is number 8 of the National Development Plan 2030. Therefore, the proposed study sought to explore the experiences of people diagnosed with malaria regarding signs and symptoms at Mhinga Village in the Vhembe District, Limpopo province.

## Research methods and design

A qualitative, exploratory, descriptive and contextual research design was used. This approach helped the researcher to understand the experiences of people diagnosed with malaria regarding the signs and symptoms at Mhinga village in the Vhembe District. This enabled the researcher to obtain responses from a wide range of participants, which allowed for participants to be themselves and provided greater accuracy to the study.

### Setting

The study was conducted at Mhinga Village, Collins Chabane Municipality, Vhembe District, Limpopo province, South Africa. Mhinga Village is near the Kruger National Park, and mosquitoes breed more in bushy areas. Furthermore, the village is situated on level terrain where, during rainy seasons, water does not flow to rivers but instead becomes stagnant for a long period, creating an ideal environment for female mosquitoes to breed. Mhinga Village is characterised by high temperatures, which limit the wearing of clothes that cover the whole body. Mhinga Village was selected as the study setting because reports had shown it has a higher malaria prevalence than other villages in the Vhembe District.

### Population

The study population consisted of individuals residing in Mhinga Village, Collins Chabane Municipality, in the Vhembe District. This included adults diagnosed with malaria aged 18–90 years of all genders, regardless of their educational background, income level, religion or marital status. The participants also spoke Tsonga, Tshivenda and English, which are the common languages in the community. The selected participants were those who were diagnosed with malaria above the age of 18 years, both males and females who reside in Mhinga village, because they were the target population and appropriate people to answer the interview questions.

### Sampling

The sampling area was Mhinga Village, which was purposively selected as it is the area with high malaria prevalence. Interviews were conducted at the study participants’ homes to help them feel comfortable. Purposive sampling was utilised by the researcher, who went to Mhinga clinic and obtained a list of diagnosed candidates, as well as their contact details and addresses.

### Sample size

The sample size was determined by data saturation. This occurred after reaching 10 participants. The researcher then added 16 more participants for in-depth, rich data, bringing the total to 26 participants for enhanced authenticity.

### Pretest

The researcher conducted a pretest by selecting two individuals at Mhinga Village who had experienced malaria, but they were not included as part of the sample. The pretest was conducted to test if participants might be overly sensitive to specific questions or hesitate to answer, and it helped to determine the level of communication skills that would be required. The pretest helped the researcher to ensure that positive results were met and to improve interviewing skills, specifically probing skills. The researcher was able to test how well the research questions worked for gathering information and ensure that the method used to gather information was correct. The researcher communicated well with the participants, tried to read their reactions, and took notes. The pretest was conducted to test if the interview guide questions were easy to understand. The pretest assisted the researcher in evaluating interviewing skills and voice recorders for quality. No alteration was made, as the pretest met the objectives.

### Data collection

A semi-structured individual face-to-face interview with interview guide questions was used to collect data from July to October 2024. The researcher encouraged the interviewees to elaborate and provide in-depth information regarding their experiences of malaria with signs and symptoms. Other questions were formed based on what the researcher perceived or observed during the interviews; the researcher was able to probe and obtain comprehensive information. Interviews were conducted in Tsonga, Tshivenda and English as preferred by the participant. Data were collected during the day on weekends, which covered those working on weekdays. Participants were reminded of their right to terminate the interview at any time when they felt it was necessary to protect their right to self-determination. The consent form was signed before obtaining data from participants. An audio recording was used. Audio recordings were transcribed into English each day by a language expert. The researcher took about 15 min – 30 min with each participant. Data saturation was reached after interviewing 10 participants, and then the researcher decided to add 16 participants to get more in-depth information. Confidentiality was ensured by not writing participants’ names and only using numbers. All participants interviewed were between the ages of 18 and 90.

### Data analysis and management

The researcher listened to the audio recorder and translated the interview, after which a language expert wrote down what was said on the audio recorder in English. After writing, the researcher read each transcription several times to get familiar with the information they had gathered and to sort ideas that were similar and those that were different. Brink, Walt and Rensburg (2018) steps were used to lead the data analysis. The researcher firstly used a ‘hands-on’ method to look at the data. This meant collecting a lot of text-based data and spending a lot of time thinking about what it all meant and how it fit with other data. The researcher read the transcript several times at this point to make sure they understood what the participants said. Secondly, codes, which were groups of letters, numbers, symbols, words or sentences, were used by the researcher to sort the data. The words of the participants were used to organise the collected data into themes and subthemes. There were five main themes, and subthemes grew out of each one. The researcher interpreted the data based on the researcher’s understanding of the experiences of those diagnosed. Lastly, an explanatory code was used to describe the different meanings that these codes came up with. These codes changed over time as the researcher learnt more about what the data meant. Electronic data were kept safe on a laptop that was protected with a password so that only the researcher could get to it. This was done to lower the risk of data being used without permission.

### Measures to ensure trustworthiness

To ensure the accuracy and credibility of the study, the researcher used four criteria: credibility, dependability, conformability and transferability. The researcher used interviews, audio recorders and field notes at the same time to increase credibility. To ensure reliability, the recordings and documentation were preserved so that the study could be conducted again with the same individuals in a comparable setting. Conformability was ensured by thoroughly listening to audio recordings to confirm interpretations, conclusions, and suggestions. Transferability was ensured by giving a detailed description, which involves the setting, participants, and methods used to collect data for other researchers to come up with the same findings and conclusions. In this way, other researchers could decide if the results were useful in other situations or if they were transferable.

### Ethical considerations

Ethical clearance to conduct this study was obtained from the University of Venda Human and Clinical Trails Research Ethics Committee on 5 April 2024. The ethical clearance number is FHS/24/PDC/01/0404. The researcher ensured quality assurance by presenting the proposal to the Department of Advanced Nursing Sciences and the Faculty of Health Sciences Higher Degrees Committee for approval. In addition, the Chief of Mhinga Village and community stakeholders gave permission. Informed consent was used to protect human rights, respecting traditional practices. Participants were allowed to choose how much sensitive information to share, and their identities were kept anonymous to ensure confidentiality.

## Results

Demographic characteristics of participants are presented in Table 1. A total of 26 participants were identified and interviewed about their experiences when they got sick with malaria at Mhinga village in the Vhembe District, Limpopo province.

**TABLE 1:** Demographic summary of participants.

Characteristics	Category	Total number
Gender	Male	15
	Female	11

**Total**		**26**
Number of episodes of malaria	Once	19
	Twice	7
**Total**		**26**
Age group	20–29	4
	30–39	5
	40–49	12
	50–59	1
	60–69	2
	70–79	1
	80–89	1
**Total**		**26**
Year of diagnosis and experience	2024	3
	2023	14
	2022	7
	2021	2
**Total**		**26**
Marital status	Single	12
	Married	10
	Widowed	2
**Total**		**26**
Employment status	Employed	10
	Unemployed	11
	Retired	3
	Student	2
**Total**		**26**

The study’s findings highlighted the experiences of those who contracted malaria in Mhinga village. Following the data collected by the researcher, two themes emerged during the interview: experiences with signs and symptoms and circumstances driving immediate consultation. These themes are presented with direct quotes from the participants alongside subthemes.

### Theme 1: Experiences with signs and symptoms

Theme 1 emerged from data indicating participants verbalising their experiences in the form of signs and symptoms, which affected their overall well-being and quality of life. Seven subthemes were identified that fall under different types of body systems, namely: central nervous system, gastrointestinal system, immune system, autonomic nervous system, central and peripheral nervous system, musculoskeletal system, and reproductive system. Each theme was discussed separately under experiences with signs and symptoms (See Table 2).

**TABLE 2:** Theme and subthemes.

Emerged themes	Subthemes	Categories
Theme 1: Experiences with signs and symptoms	1.1 Central nervous system	Constant headache and dizziness Confusion General body pain Pins and needles sensation Joint pain Thirst
	1.2 Gastrointestinal system	Nausea and vomiting Abdominal pain Food intolerance Taste impairment Diarrhoea
	1.3 Immune system	Fever and chills Shivering
	1.4 Autonomic nervous system	Sweating
	1.5 Central and peripheral nervous system	Inability to sleep
	1.6 Musculoskeletal system	Fatigue
	1.7 Reproductive system	Low libido
Theme 2: Circumstances driving immediate consultation	2.1 Experience impacted by health information	
	2.2 Previous experience	
	2.3 Family diagnosis	
	2.4 Family motivation	

#### Subtheme 1.1: Central nervous system

The study’s findings revealed that certain symptoms affected the central nervous system, such as constant headache, dizziness, confusion, general body pain, pins and needles sensation, joint pain, and thirst. As a result, one of the first experienced symptoms reported after being diagnosed with malaria was continuous headache and dizziness. The participants took medication from nearby spaza shops and those available in the house to cope with the headache. However, the participants indicated that they would feel better for a brief period of time:

‘I decided to go to the spaza shop and get some painkillers, which I took, and it helped me to feel better, and the headache would stop for a few hours.’ (P1, F, 35 years)

‘I first had a sharp headache which felt like my head was spinning when I woke up in the morning.’ (P3, M, 45 years)

Participants went on to describe feelings of confusion and mentioned it felt exacerbated by physical touch and extreme pain, both symptoms happening at once, and not knowing what was happening. The general body pain was described as extremely worsened by physical touch, which raised fears of the unknown:

‘I would feel confused, especially when someone touches my body, and would feel as if I am crazy at times because it was too painful, and I could not explain what was happening inside my head.’ (P2, F, 48 years)

‘My body was too painful, especially when I touch each part of the body, I would not even know where the pain is exactly.’ (P3, M, 45 years)

A few participants reported experiencing pins and needle sensations, which were described as an uncomfortable tingling or prickling from and to the legs, worsened by body pains, and occurring throughout the day. The study found that the participants drank a lot of water during infection:

‘My legs felt like there was something, as if they were freezing, like something was moving around my legs to the top.’ (P17, F, 45 years)

‘We were always asking for water, we drank a lot of it on the road.’ (P17, F, 45 years)

#### Subtheme 1.2: Gastrointestinal system

Symptoms affecting the gastrointestinal system were nausea and vomiting, abdominal pain, food intolerance, taste impairment and diarrhoea. Most participants frequently reported experiencing nausea and vomiting, which was found to happen throughout the initial days of the infection. The findings revealed that this symptom had caused food intolerance, as vomiting was constant with each food intake. Abdominal pains were expressed to be triggered after eating food and were indescribable according to participants because of multiple symptoms occurring at once, as evidenced by the following:

‘Vomiting at times and nausea prevented me from eating food.’ (P2, F, 48 years)

‘I did not like eating. When I ate food, it felt tasteless, honestly.’ (P20, F, 48 years)

‘I had abdominal pains, especially when I ate food.’ (P10, M, 24 years)

‘Yes, when I was sick of malaria, I had a running stomach. I would drink water and mageu to manage.’ (P23, M, 49 years)

#### Subtheme 1.3: Immune system

The immune system as a subtheme emerged from the participants’ responses, which revealed that fever and chills were among the most commonly experienced symptoms in the first few days of illness:

‘My body felt hot on touch, and I was feeling cold. Around 15h00, I would put on a lot of blankets to help with the cold for three days until I decided to visit the clinic.’ (P2, F, 48 years)

Shivering was described to occur with chills, headache and dizziness, and it was expressed to be worsened by being in a shadowed area:

‘I was feeling cold, shivering. It happened the first day, I was shivering, staying under the sun.’ (P20, F, 48 years)

#### Subtheme 1.4: Autonomic nervous system

Participants mentioned that sweating had occurred together with fever and chills. The findings revealed that this symptom was described to take place in the morning and night and would be triggered by feeling hot and using blankets at night to cover up:

‘I felt weak when I slept, I was very sweaty, like someone poured water on me, and my blanket would be very wet.’ (P24, F, 25 years)

#### Subtheme 1.5: Central and peripheral nervous system

The study’s findings have shown that there was an inability to sleep. The struggle with sleep occurred during the night because of heat and was worsened by body pain, as evidenced by the following quotations:

‘It was not easy to sleep at night due to body pains, but during the day I would sleep a bit but the body was too painful.’ (P2, F, 48 years)

#### Subtheme 1.6: Musculoskeletal system

The study’s findings revealed that most participants experienced fatigue. Participants reported struggles with movement from the bed, taking care of oneself, daily chores, and difficulties walking short distances and expressed this to have occurred throughout the time of sickness:

‘I even collapsed at home after walking a few steps, feeling very tired, I was not able to do anything, even walking was difficult, to bathe and eat, it was as if I was drugged when walking. I would sleep in bed the whole five days till the day I collapsed. I really thought I was about to die. I thank God I didn’t die.’ (P10, M, 24 years)

‘I was weak, my friends and daughter were helping me to make food, clean, and wash dishes.’ (P24, F, 25 years)

#### Subtheme 1.7: Reproductive system

This subtheme emerged when a participant indicated having experienced low libido during the period of sickness. The participant expressed experiencing decreased sexual desire. The participant reported that this symptom was aggravated by body weakness:

‘I was having low interest in sex or anything intimate.’ (P21, M, 45 years)

### Theme 2: Circumstances driving immediate consultation

The study’s findings suggested that certain situations and events led participants to avoid procrastination and consult immediately. The participants indicated that the delay in consultation was mainly because of ignorance and a lack of funds. However, not all participants delayed consultation. There were certain factors that made participants seek treatment, and four subthemes were revealed: previous education, previous experience, family diagnosis and family motivation:

‘My wife advised me to go to the clinic since our children were diagnosed with malaria the same week. I decided to wait a bit until symptoms were constant, then I was taken to the clinic when symptoms got worse.’(P5, M, 47 years)

‘Then we stayed a bit because we did not have money at the time. I remember we had to call someone to send us money then my girl child went to the spaza shop to get cash in order to pay for transport, then we went to get a bus, we got it then arrived at the clinic.’ (P16, F, 45 years)

#### Subtheme 2.1: Experience impacted by health information

The study revealed that some participants sought medical advice within 6 h of experiencing symptoms. This prompt action was largely influenced by the health information shared by Dr Phophi Ramathuba, the Member of the Executive Council (MEC), who educated the public during visits to health facilities, as expressed by the participants:

‘I first had a sharp headache which felt like my head was spinning when I woke up in the morning, the headache was accompanied by dizziness, it happened from 7 am to 13h00 until I decided to go to the clinic the same day because I knew that this must be a sign of malaria as I had travelled from Mozambique the previous three days before the onset of symptoms and I knew the malaria symptoms and its causes considering that Mozambique is also a malaria area.’ (P3, M, 45 years)

‘The time Dr Phophi Ramathuba came, we were taught about it.’ (P3, M, 45 years)

#### Subtheme 2.2: Previous experience

This subtheme emerged as a result of participants revealing that they have experienced malaria more than once. These participants expressed that symptoms were the same in both occurrences, which motivated them to seek help immediately to avoid complications. Because of their previous experience and those of family members, they sought medical attention immediately:

‘We were infected twice.’ (P17, F, 45 years)

‘It was around 2019, the first time when I went to the clinic, then they didn’t give me anything, they said the pills were out of stock.’ (P25, M, 38 years)

#### Subtheme 2.3: Family diagnosis

According to the study’s findings, more than one family member was infected simultaneously. It was stated that seeking immediate consultation was because of the illness of the young ones having similar symptoms to those of their parent, which led to the parent visiting the clinic and getting tested:

‘I told them how I was feeling and asked them to check me for malaria because my kid was diagnosed positive the same week.’ (P10, M, 24 years)

‘When I went to the clinic, it was because my son was not well. I was also sick, so I decided to get checked too, since my body was unwell. I followed my son where they were testing him, and they asked what I was feeling. Then, I told them that I had been struggling to sleep.’ (P17, F, 45 years)

#### Subtheme 2.4: Family motivation

The participants expressed that their family members had encouraged them to consult after observing the constant worsening of symptoms. Some of the participants showed that they were diagnosed for the second time and thus had similar symptoms to those they suffered during their first episode. Hence, they immediately sought medical attention:

‘I had a headache for two days, which was accompanied by dizziness. My wife advised me to go to the clinic since our children were diagnosed with malaria the same week. I decided to wait a bit until symptoms were constant, then I was taken to the clinic when symptoms got worse.’ (P5, M, 47 years)

‘It was day four or five when I woke up feeling weak. I even collapsed at home after walking a few steps. My mother checked and helped me and then told me to bathe and go to the clinic.’ (P11, M, 25 years)

## Contributions

This study highlights the necessity of timely consultations to prevent malaria complications, as malaria is a serious condition. It also advocates for community awareness campaigns to encourage prompt action when signs and symptoms of malaria arise.

## Discussions

The majority of the participants reported persistent headaches and dizziness, followed by exhaustion, bodily discomfort, fever and chills. The symptoms and their causes were clearly stated. The study found that symptoms appeared in the first few days of illness and worsened with time. Some people experienced the same symptoms but reported minor distress. Touch aggravated the terrible anguish, causing fear of the unknown. An intense, whirling headache was described. The findings are consistent with Hijazi et al. (2022), who reported headache, dizziness and fatigue. These symptoms would occur simultaneously and worsen with movement. Participants mentioned visiting spaza shops to buy medication to alleviate symptoms. Other studies in Uganda’s Batwa in Kanungu District found that the first participant also suffered headache, body discomfort, fever and chills, and bought medication from neighbouring pharmacies but did not feel better (Namanya et al. 2023). World Health Organization (2023) reports that weakness, body pains, fever and chills begin a few days after the bite, and symptoms might be severe or mild. Participants reported sleeping all day because of body pain and fatigue; walking short distances was difficult, and staying in the sun to keep warm helped them cope with fever and chills. Namanya et al. (2023) found that the sixth Batwa participant in their survey had experienced excessive temperatures and wanted to sit by a fireplace or in the sun. Most participants said these problems impacted their daily lives, as they required help from parents, spouses, children, and friends. Bria, Yeh and Bedingfield (2021) reported that Indonesians diagnose malaria with fever and shivering.

Based on the findings, some participants associated these symptoms with Coronavirus and influenza. Khew, Akbar and Mohd-Assaad (2023) showed that malaria and influenza may have identical symptoms, making COVID-19 and malaria difficult to identify. According to Wilairatana et al. (2022), influenza and malaria are coendemic in many places and difficult to identify based on clinical criteria. For instance, a study by Namanya et al. (2023) reported that a 28-year-old Nigerian student had contracted malaria 5 days after arriving in the United Kingdom. According to Harp et al. (2021), malaria is endemic in Mozambique, putting the entire population at risk, which further cements that malaria is a coendemic disease. Sweating has been linked to moving from shaded to sunny places. The Centers for Disease Control (2023) states that fever is a typical defence mechanism reaction to the disease. Participants also had trouble eating and feeling full because shivering caused them to lose their appetite. Namanya et al. (2023) found that the second participant had a decreased appetite, preventing them from eating their favourite food. Many individuals avoid eating because of nausea and vomiting. This symptom persisted with every meal, regardless of quantity. A 28-year-old woman with nausea and vomiting after eating was diagnosed with post-infectious cerebellitis related to Plasmodium falciparum malaria (Hijazi et al. 2022).

Dr Phophi Ramathuba, who was the MEC for Health in Limpopo province at the time, was recognised as a key source of malaria education during her visits to healthcare facilities. The education included malaria causes and the transmission cycle. Participants expressed that symptoms were the same in both episodes, which motivated them to seek help immediately to avoid complications. Ribeiro, Havik and Craveiro (2021) indicated that knowledge and experiences of symptoms trigger healthcare-seeking behaviours, which is presumably the reason the community members consulted immediately before symptoms worsened. Dijkman et al. (2022) reported that factors influencing family involvement in treatment decision-making involve the medical history and genetics. In this study, participants expressed that parents and spouses encouraged them to consult after observing the constant worsening of symptoms. Samari et al. (2022) indicated that people who are struggling and unwell, and who share their challenges with family and friends, are motivated and more inclined to seek treatment.

### Strengths and limitations

The findings of the study are essential for increasing knowledge and raising awareness of the signs and symptoms of malaria at Mhinga village in the Vhembe District, Limpopo province. The study was limited to one village in Vhembe District, Limpopo province. Hence, the findings cannot be generalised to the other settings because only one village was studied. In this study, only people aged 18–90 years were interviewed; however, from interviews with their parents, the findings revealed that children are also getting malaria. The scope of the study was to determine the experiences of people diagnosed with malaria; the sampling method caused difficulty as the information of patients was at times incomplete, incorrect and missing. Other participants were unable to be located via phone.

### Recommendations

The department needs to provide bed nets, as the Mhinga community has people who are mostly living in poverty. Additionally, some people live in very small RDP houses; they live in large numbers. Thus, education on the impact of overcrowding on households should also be considered. There is a need for waste removal at Mhinga village to reduce breeding sites. Further research is required to explore the experiences of children under the age of 18 in chosen, affected areas who have been diagnosed with malaria.

## Conclusions

Malaria poses a significant health burden in South Africa, leading to numerous infections and fatalities, particularly in regions with high temperatures. The study found that participants exhibited various signs and symptoms of malaria, influenced by different bodily systems. Several factors influenced participants to seek treatment, including prior education, personal experiences, family diagnoses and family motivation. Therefore, it is crucial to raise health awareness to encourage immediate consultation when individuals experience signs and symptoms of malaria.
